# Public awareness, knowledge and attitudes towards dyslexia in China

**DOI:** 10.1007/s11881-026-00377-y

**Published:** 2026-06-22

**Authors:** Shuyu Chen, Yaomin Huang, Juhayna Taha

**Affiliations:** 1 Academic Unit of Human Communication, Learning, and Development, Faculty of Education, The University of Hong Kong, Hong Kong, China; 2 Faculty of Education, University of Cambridge, Cambridge, United Kingdom; 3 Department of Psychology and Human Development, Institute of Education, University College London, London, United Kingdom

**Keywords:** Public awareness, Attitudes, Knowledge, Dyslexia, China, Survey

## Abstract

This study is the first to examine awareness, knowledge, and attitudes towards dyslexia among the general public in Mainland China. Using an online survey, we collected data on (a) demographics, (b) awareness of dyslexia and other common neurodevelopmental conditions, (c) knowledge of dyslexia causes, symptoms, functional impact, and assessment/intervention, and (d) attitudes towards dyslexia. A total of 1,008 adults from across all major regions of China completed the suvey. Around 70% reported having heard of dyslexia, lower than awareness of autism and ADHD, but higher than that of developmental language disorder. Respondents answered 49% of knowledge items correctly and demonstrated greater knowledge of dyslexia symptoms, followed by functional impact and causes, with weaker knowledge of dyslexia assessment and intervention. Dyslexia awareness and knowledge were higher among younger adults, females, urban residents, non-parents, and those with higher education and income, with some variation across regions. Attitudes towards dyslexia were generally positive, following similar demographic patterns. Although greater awareness was associated with higher levels of knowledge, only dyslexia knowledge uniquely predicted attitudes towards dyslexia after controlling for demographic factors. These findings highlight the need for culturally relevant, awareness-raising campaigns that promote a more accurate understanding of dyslexia. The findings should be interpreted in light of the limitations linked to sampling bias and methods of data collection. Future studies should include the voices of individuals with dyslexia to better understand how social and cultural factors in China influence their lived experiences across development.

**Supplementary Information:**

The online version contains supplementary material available at https://doi.org/10.1007/s11881-026-00377-y.

## Introduction

In China, up to 9.7% of primary school children have dyslexia (Cai et al., 2020; Chan et al., 2007; Feng et al., 2022; Lin et al., 2020). Dyslexia is a specific learning disorder characterised by slow and inaccurate word reading and poor spelling that persists despite effective instruction, varies across orthographies, and occurs along a continuum of severity. Dyslexia may be associated with secondary difficulties in reading comprehension, reduced reading and writing experiences, which may impede language and knowledge development and academic attainment (International Dyslexia Association, 2025). Children with dyslexia are at an increased risk of academic underachievement, falling behind their peers and dropping out early from school (Zhou, 2022). They also have a significantly lower quality of life (Huang et al., 2020; Zou et al., 2022). Studies using parent reports indicate that some children with dyslexia exhibit greater emotional and behavioural problems, and lower prosocial behaviours relative to their typically developing peers (Huang et al., 2020; Feng et al., 2024). These children report greater levels of anxiety, depression, low self-esteem, and exposure to bullying and school-related stress (Xiao et al., 2023; Wang, 2021; Chung et al., 2024; Feng et al., 2024). Although typically identified in childhood, dyslexia is a lifelong condition that persists into adolescence and adulthood (Shaywitz, 1999; Lohvansuu et al., 2021; Nergård-Nilssen & Hulme, 2014; Reis et al., 2020). Adults with dyslexia are at greater risk of unemployment, underemployment, and lower income compared to their peers (de Beer et al., 2014; Gerber, 2012). Many also experience anxiety and depression, which further restrict their participation in the workforce (Kirby & Gibbon, 2018). Given the prevalence and long-term negative impact of dyslexia, it is considered a public health issue (Sanfilippo et al., 2020).

From the social model of disability perspective, the challenges and barriers faced by individuals with dyslexia are not solely the result of reading impairments but can be exacerbated by negative attitudes of society, rigid cultural practices and norms around literacy and disability (Riddick, 2001). Therefore, increasing public awareness is critical to reducing stigma, promoting social inclusion, and improving early identification and support for individuals with disabilities, including those with dyslexia. To do so, it is essential to identify gaps in public knowledge, beliefs, and attitudes, which can inform the design of targeted education and awareness initiatives (World Health Organization, 2011, p. 267).

No empirical data about public understanding and perceptions of dyslexia in China is available. In a recent review, Zhang et al. (2023) highlighted that despite advances in research on the behavioural, cognitive, and neurobiological markers of dyslexia in Chinese, there is a lack of knowledge of dyslexia among parents and teachers. They also highlighted the importance of improving awareness of dyslexia among the public in China. In this study, we address this need by evaluating the general public’s awareness, knowledge, and attitudes towards dyslexia in China. We operationalise *awareness* as familiarity with the term dyslexia, *knowledge* as understanding of dyslexia, including its features, causes, and impact, and *attitudes* as a complex construct comprising affective, cognitive, and behavioural components that influence how individuals think, feel, and act towards children and adults with dyslexia (McCaughey & Strohmer, 2005; Findler et al., 2007).

### Public awareness, knowledge and attitudes towards dyslexia across different countries

Research shows that public awareness, knowledge, and attitudes towards dyslexia vary across countries. Dyslexia awareness and knowledge appear to be generally good in developed countries such as the United States (US) and the United Kingdom (UK; Castillo & Gilger, 2018; Furnham, 2013). For instance, Castillo & Gilger (2018) examined perceptions of dyslexia among 623 participants in the US and found that 97% had heard of dyslexia and 91% reported knowing something about it. In contrast, lower awareness has been reported in other contexts. Duranović et al. (2011) surveyed 1,701 participants across five Balkan countries and found that, on average, only 54% had heard of dyslexia, and around 42.5% were classified as having some basic knowledge. Awareness and knowledge were highest in Croatia (~ 88% and ~ 73%) and Bulgaria (~ 72% and ~ 66%), followed by Montenegro (~ 70% and ~ 45%), and lowest in Bosnia and Herzegovina (~ 48% and ~ 35%) and Kosovo (~ 23% and ~ 18%). To our knowledge, public awareness and knowledge of dyslexia in non-Western contexts are under-explored, although studies with specific groups (e.g., teachers, students, doctors) in countries such as Nigeria, Ghana, Saudi Arabia, South Korea, and Pakistan suggest limited dyslexia knowledge (Ajoku-Christopher, 2012; Abed & Shackelford, 2022; Lee & Shin, 2019; Maahin et al., 2025).

Across studies, the public demonstrated the strongest knowledge of the observable behavioural characteristics of dyslexia, such as difficulties in reading, writing, and spelling (Castillo & Gilger, 2018; Duranović et al., 2011; Furnham, 2013). Knowledge of other aspects was inconsistent. Fewer than 40% of UK participants identified dyslexia as a neurobiological condition (Furnham, 2013), whereas biological and genetic causes were more commonly recognised in the US (Castillo & Gilger, 2018). However, misconceptions were prevalent, particularly associating dyslexia with vision problems across all three studies. Some misconceptions, such as attributing dyslexia to mental health problems, low intelligence, or neurological damage (e.g., stroke), were primarily observed in the Balkan study (Duranović et al., 2011). Knowledge of interventions also appeared to be limited. In the US, many participants believed that children could control features of their dyslexia (Castillo & Gilger, 2018), while in the UK, nearly one-third believed that dyslexia could be cured through intensive training (Furnham, 2013). In the Balkan countries, limited awareness of available services and support was reported (Duranović et al., 2011). There is also evidence that public knowledge of the long-term consequences of dyslexia is limited, with participants underestimating its impact on academic and life outcomes (Castillo & Gilger, 2018).

Much less empirical evidence is available on public attitudes towards dyslexia. Despite the knowledge gaps outlined above, attitudes in the US and UK studies were generally positive, with the majority of participants endorsing teacher training and the provision of educational and workplace support (Castillo & Gilger, 2018; Furnham, 2013). As noted by Furnham (2013), positive public attitudes may coexist with limited or inaccurate knowledge of dyslexia. Drawing on a review of policy and programme evaluations, Fisher & Purcal (2017) report that information and awareness-raising initiatives can increase knowledge and understanding of disability but that evidence for attitude change is inconsistent.

Across countries, awareness and knowledge were associated with demographic factors such as age, gender, education, and ethnicity (Castillo & Gilger, 2018; Duranović et al., 2011; Furnham, 2013). In the Balkans, higher awareness and knowledge were observed among younger adults, women, individuals with higher educational attainment, and those in professional roles (Duranović et al., 2011). In the US study, Asian participants were more likely to view dyslexia as less severe and more controllable than White participants, which may contribute to delayed recognition and under-identification of dyslexia (Castillo & Gilger, 2018).

Importantly, cross-study differences in awareness and knowledge should be interpreted with caution. Higher awareness and knowledge in Croatia and Bulgaria were based on samples with higher levels of education and professional employment, whereas lower awareness in Bosnia and Herzegovina and Kosovo was observed in samples with lower educational attainment and higher proportions of unemployed or lower-skilled participants (Duranović et al., 2011). Similarly, the UK sample was biased towards younger, better-educated, and higher social-class participants, with 66% reporting personal familiarity with dyslexia (Furnham, 2013). There are also methodological differences between the studies. While the Balkan and UK studies relied on face-to-face convenience sampling, the US study recruited a sample via an online Amazon Mechanical Turk platform that is known to over-represent younger and more educated respondents (Cheung et al., 2017; Huff & Tingley, 2015; Walters et al., 2018). In addition, dyslexia knowledge was operationalised differently across studies, with the Balkan study applying a more stringent categorical classification of “basic knowledge”, potentially contributing to lower reported knowledge levels relative to the US and UK.

Taken together, differences in demographic composition, methodological approaches, and broader contextual factors can influence public awareness and understanding of dyslexia. Hence, it is important to understand the country-specific factors that influence dyslexia awareness and understanding. In the next section, we discuss such factors in the Chinese context.

### Awareness, knowledge and attitudes towards dyslexia in China

For a long time, it was believed that dyslexia either did not exist or was extremely rare in Chinese (Caravolas, 2005), until Stevenson et al. (1982) first documented its presence in the language. Later, epidemiological studies estimated that 3.9% to 9.7% of primary school-age children in China have dyslexia (Cai et al., 2020; Chan et al., 2007; Feng et al., 2022; Lin et al., 2020). These studies identified risk factors for dyslexia in China, including being male, lacking learning habits, reduced home literacy environments, low parental education, and limited reading experiences. The variations in dyslexia prevalence in China reflect the inconsistencies in definitions, diagnostic criteria, and assessment tools used in the different studies. To date, there is no single standardised test for dyslexia in China (McBride et al., 2018; Xia et al., 2024), placing many children at risk of under-identification.

There is a heavy influence of Confucianism in China, an ancient philosophy that underpins many aspects of daily life and emphasises social harmony, ethical behaviour, and family relationships. Confucianism places a high value on education, which is viewed as a family responsibility. Chinese parents prioritise educational success (Huang & Gove, 2015) and tend to have strong control and high expectations for their children to excel academically (Baumrind, 1971; Maccoby & Martin, 1983; Chao & Tseng, 2002). They also place greater emphasis on effort than ability in educational activities (Rao et al., 2000). Perseverance and overcoming difficulties are regarded as key virtues (Li, 2012). As a result, low achievement may be seen as shameful and embarrassing to the family (Chen et al., 1997). These cultural beliefs may contribute to the under-detection, invisibility, and limited public awareness of dyslexia, as reading difficulties may be attributed to laziness, lack of effort, or poor discipline rather than to neurodevelopmental conditions (McBride et al., 2018). For example, Chinese parents of children with dyslexia have reported feelings of sorrow, self-blame, and a determination for both themselves and their children to work harder to overcome dyslexia (Poon-McBrayer & McBrayer, 2014). These beliefs may delay identification, limit access to support, and reinforce stigma towards both children with dyslexia and their families.

China has introduced several laws and policies related to disability. The Law of the People’s Republic of China on the Protection of Disabled Persons, established in 1990 and amended in 2008, guarantees equal rights to people with disabilities, particularly in cultural participation, including accessible provisions (e.g., sign language programmes and Braille resources; National People’s Congress, 2008). However, the law mainly refers to physical, visual, and mental impairments. It promotes inclusion through the lens of productivity and perseverance, which encourages individuals to demonstrate “a spirit of tireless self-improvement” (Dauncey, 2020, p. 13). This reflects a broader cultural view that equates disability inclusion with usefulness and achievement. Although progress has been made, ongoing barriers, including stigma, limited access to services, and restricted social engagement, continue to hinder social participation. The emphasis on visible disabilities and individual perseverance may contribute to the invisibility and misunderstanding of neurodevelopmental conditions such as dyslexia.

The Ministry of Education of the People’s Republic of China published several policies and reports to advance special and inclusive education. Although these initiatives may enhance visibility and awareness of disability, they did not explicitly refer to dyslexia. Additionally, information about dyslexia has increasingly become available online through blogs, discussion groups, and posts on popular social media platforms. However, the quality and accuracy of this content vary considerably. There are also a number of centres, clinics, and organisations focusing on learning difficulties, including dyslexia. These centres appear to be largely concentrated in major cities such as Beijing, Shanghai, Shenzhen, and Guangzhou.

Data on the public awareness of dyslexia in China is lacking. Yin et al. (2020) assessed dyslexia knowledge among 516 early literacy teachers across developed and less developed regions in China. On average, teachers answered 53.9% (~ 16/29) of the survey statements correctly, a figure considerably lower than that reported for teachers in the US (87.7%), UK (76.7%), and Australia (71%; Washburn et al., 2017; Serry & Hammond, 2015). Teachers showed basic knowledge of dyslexia symptoms, such as difficulties with decoding, spelling, and reading fluency. However, the understanding of dyslexia causes was limited. Over 60% did not recognise the neurological basis of dyslexia, misunderstood it as being caused by a poor home literacy environment, and were unaware that it is a lifelong condition. Although many were familiar with evidence-based instructional strategies, such as structured instruction and repeated reading, misconceptions about unproven interventions were widespread. Over 95% believed eye-tracking exercises were practical, and more than 80% were unaware that coloured overlays are not supported by evidence. Importantly, regional disparities were evident, with teachers in less developed areas scoring consistently lower across all domains compared to teachers from more developed regions. This points to systemic inequities in access to training and professional development that disproportionately affect teachers in less developed areas.

### Current Study

Several characteristics of Chinese society, such as underdeveloped dyslexia services, the influence of Confucian values, and disability policies that may overlook dyslexia or attribute it to laziness or lack of effort, create a context in which dyslexia is under-recognised and poorly understood. This is reflected in findings by Yin et al. (2020), which showed limited knowledge of dyslexia among Chinese teachers. These findings are likely to be mirrored among the public. The public plays a significant role in influencing the experiences of individuals with disabilities, including those with dyslexia. This study aims to assess public awareness, knowledge, and attitudes towards dyslexia in China. Specifically, we ask:

1. What is the level of public awareness of dyslexia in Mainland China?
2. What is the level of public knowledge about dyslexia?
3. What are the public attitudes toward dyslexia in China?
4. How do public awareness, knowledge, and attitudes relate to each other?

Due to the previously long-standing belief that dyslexia does not exist in the Chinese language (Caravolas, 2005), we predict that public awareness of dyslexia in China will be low. Similar to Yin et al.’s (2020) findings, we also expect that public knowledge will be limited, with better recognition of dyslexia’s behavioural symptoms, such as reading and spelling difficulties, but less understanding of dyslexia’s causes, treatment and long-term impact (Furnham, 2013; Castillo & Gilger, 2018). We also predict that public attitudes will be relatively negative due to Chinese cultural values that are rooted in Confucianism, as well as the limited awareness of dyslexia. In line with the previous studies, we predict that awareness, knowledge, and attitudes will vary across demographic groups such as age, gender, educational levels, socio-economic status, as well as region (Duranović et al., 2011; Furnham, 2013; Castillo & Gilger, 2018; Yin et al., 2020). Given the limited and inconsistent evidence on the relationship between awareness, knowledge, and attitudes (e.g., Furnham, 2013; Fisher & Purcal, 2017), no directional hypothesis was specified for their association.

## Method

This study received approval from the research ethics committee at MA Special and Inclusive Education (Specific Learning Difficulties) at University College London and was conducted in line with the British Psychological Society Code of Ethics and Conduct.

### Participants

To be eligible for the study, participants were required to be aged 18 and above and residing in Mainland China.

### Survey design

The English version of the survey is provided in Appendix A. The first section consisted of nine questions and collected demographic information about the participants, such as age group, gender, region, and educational and professional backgrounds. The second section consisted of six questions and assessed participants’ awareness and knowledge of dyslexia. The first multiple-choice question asked about whether participants had heard of dyslexia or other common neurodevelopmental conditions (e.g., autism, hearing loss, ADHD, developmental language disorder), with the options “Yes”, “No”, and “Unsure”. If “Yes” or “Unsure”, participants were asked to indicate the source of dyslexia awareness. The third question contained a checklist of 38 statements that assessed dyslexia knowledge: 17 statements were adapted from Yin et al. (2020; items 2, 3, 5-11, 14, 15, 17, 18, 29-31, 33), ten statements were adapted from Castillo & Gilger (2018; items 1, 4, 19-23, 26, 32, and 36), and the remaining 11 statements (items 12, 13, 16, 24, 25, 27, 28, 34, 35, 37, and 38) were developed by the first and last authors to address culturally specific misconceptions about dyslexia in the Chinese context. The statements covered four areas of knowledge: causes and prevalence, symptoms, functional impact and outcomes, and assessment and treatment (see Table 3 for a list of statements for each area). Some of these statements were correct, whereas others represented common misconceptions about dyslexia. The statements appeared in a randomised order. Participants were required to respond to each statement with “True”, “False” or “I do not know”. The remaining three questions in this section were multiple-choice items assessing participants’ knowledge of which professionals are involved in the assessment and treatment of dyslexia. The third section included six Likert-scale statements designed to measure participants’ attitudes towards dyslexia. For each statement, participants rated their agreement on a scale from ‘Strongly disagree’ to ‘Strongly agree’.

### Data collection

The survey was created in Simplified Chinese and shared using the Qualtrics Software XM Online Platform. The Chinese version of the survey was piloted with 10 individuals (mean age = 25.67 years), who completed it and provided feedback on the clarity of the items, and only minor wording changes were made accordingly. Data from the pilot participants were not included in the final analysis. Participants were recruited using convenience sampling, primarily through advertisements posted on popular Chinese social media platforms such as WeChat. We also employed snowball sampling, where participants were encouraged to share the survey link with others in their networks. These approaches enabled us to reach individuals across different regions (Leighton et al., 2021). Data were collected in two waves. The first wave took place between February and April 2025. However, there was a lack of responses from some regions of China. A second wave of data collection was therefore conducted between November and December 2025 to improve regional representation.

### Data analysis

Data were analysed using IBM SPSS (Version 29.0). To address the first research question, descriptive statistics summarised the percentage of participants who had, had not, or were unsure whether they had heard of dyslexia (and other neurodevelopmental conditions), as well as sources of information. For clarity, “No” and “Unsure” responses were combined into one category. Seven chi-square tests examined differences in dyslexia awareness across demographic groups. Post-hoc comparisons with Bonferroni correction adjusted the significance threshold to 0.0071 (Sharpe, 2015). Fisher’s Exact Test was used when more than 25% of expected cell counts were below 5.

For the second research question, each correct response on the 38-item checklist was scored as 1, and incorrect/unsure responses as 0 (maximum score = 38). Internal reliability was good (α = 0.90; subscales: causes/prevalence α = 0.73, symptoms α = 0.83, functional impact α = 0.74, assessment/treatment α = 0.59). Total and subscale accuracy scores (percentage correct) were calculated and compared using a one-way repeated-measures ANOVA (Huynh-Feldt correction where sphericity was violated), with Bonferroni-adjusted post hoc tests. Group differences were examined using t-tests (gender, area, parental status) and one-way ANOVAs (age, geographical region, education, income), with Bonferroni-adjusted *p*-value = 0.0071. Tukey HSD tests followed significant ANOVAs.

For the third research question, negatively worded attitude items were reverse-coded so higher scores reflected more positive attitudes. Scores were summed across six items (range 6–30; α = 0.61). Mean scores and SDs were calculated by demographic group and compared using the same t-test/ANOVA approach and Bonferroni threshold. Although normality was violated (Shapiro-Wilk, *p* <.05), the absence of extreme outliers and large sample size justified parametric tests under the Central Limit Theorem. Welch’s corrections were reported where homogeneity of variance was violated.

To address the fourth research question, independent-samples t-tests and Pearson’s correlations were conducted. Hierarchical stepwise regression analyses then examined associations between dyslexia awareness, knowledge, and attitudes, with demographic covariates (as informed by significant effects identified for previous research questions) entered in Step 1 and main predictors in Step 2.

## Results

### Respondents

A total of 1,284 respondents accessed the survey. We excluded eight respondents who did not give consent to participate, and 251 who did not complete the entire survey. Using Qualtrics’ fraud detection metrics, we further excluded six respondents suspected of being bots (identified by a Recaptcha_Score < 0.5, suggesting a high likelihood of an automated response), four who submitted multiple responses (as captured by both the RelevantID_Duplicate and RelevantID_DuplicateScore fields which track repeated submissions from the same browser and device), and seven who provided fraudulent responses (identified by a RelevantIDFraudScore of 30 or above which captures low quality responses based on inconsistencies such as IP address, browser configuration, and location).

Table 1 presents the demographic characteristics of the final sample, which consisted of 1,008 respondents.

**Table 1:** Respondents’ demographic characteristics

Variable	Frequency	Percentage	Census
**Age (years)**	*n*	%	%
18–29	350	34.7	10.66
30–39	209	20.7	15.63
40–49	263	26.1	13.74
50–59	150	14.9	16.70
60+	36	3.6	21.10
**Gender**			
Female	581	57.6	48.96
Male	419	41.9	51.04
Prefer not to say	8	0.8	-
**Area of residence**			
Rural	127	12.6	33.84
Urban	881	87.4	66.16
**Geographical region**			
East China	372	36.9	-
North China	100	9.9	-
Northeast China	188	18.7	-
Northwestern China	153	15.2	-
South Central China	159	15.8	-
Southwestern China	36	3.6	-
**Education level**			
Primary school	35	3.5	24.87
Secondary/vocational school	207	20.5	52.00
College/bachelor’s degree	629	62.4	18.25
Postgraduate degrees	133	13.2	0.95
Others	4	0.4	
**Income level**			
Low income	248	24.6	40.00
Middle income	360	35.7	40.00
High income	202	20.0	20.00
Prefer not to say	198	19.6	-
**Parental status**			
Non-parents	420	41.7	-
Parents	588	58.3	-
**Children under 18?** (***n*** = 588)			
No	253	43.0	-
Yes	335	57.0	-

As shown in Table 1, the sample included representation of all demographic categories. However, compared to national census data in China (National Bureau of Statistics of China, 2024), some demographic groups were either over-represented or under-represented. In terms of age groups, the distribution was skewed towards younger adults: 34.7% of respondents were aged 18–29 and 26.1% were aged 40–49, both notably over-represented compared to national census data. In contrast, older age groups were under-represented, comprising only 18.5% of the sample. Compared to the balanced gender distribution reported in census data, females were over-represented, accounting for 57.6% of the sample. In line with national data, most respondents resided in urban areas, comprising 87.4% of the sample.

Following Ni et al.’s (2021) approach, Chinese provinces were grouped into six major geographical regions. This was also consistent with the classification used by the National Bureau of Statistics of China (2024), which groups provinces with similar economic and demographic profiles. Specifically, Jiangxi, Shandong, Anhui, Fujian, Jiangsu, Zhejiang, and Shanghai were grouped into East China. Neimenggu, Hebei, Shanxi, Beijing, and Tianjin were grouped into North China. Heilongjiang, Jilin, and Liaoning were in Northeast China. Xinjiang, Qinghai, Gansu, Shaanxi, and Ningxia were in Northwestern China. Guangxi, Hunan, Hubei, Guangdong, Henan, and Hainan were in South Central China. Xizang, Sichuan, Yunnan, Guizhou, and Chongqing were in Southwestern China. In the current sample, respondents represented all six major regions. The educational levels of the respondents were higher than the national average, with 62.4% holding a junior college or bachelor’s degree and 13.2% a postgraduate degree, whereas only 3.5% reported having primary education, and none reported having no formal schooling. Individuals with low-income levels (24.6%) were under-represented in the current sample, whereas the proportions of those with middle and high income were generally consistent with national averages. Also, 58.3% of respondents reported having children, most (57%) of whom had at least one child under 18 years old. Respondents represented all major work sectors, with 14.3% working in medical, health, or social care, another 20.4% being students, and 15.9% working in education. However, less than 2% were employed in technology or IT, travel, sport, or leisure, and creative arts or media.

### Dyslexia awareness

**Fig. 1:**
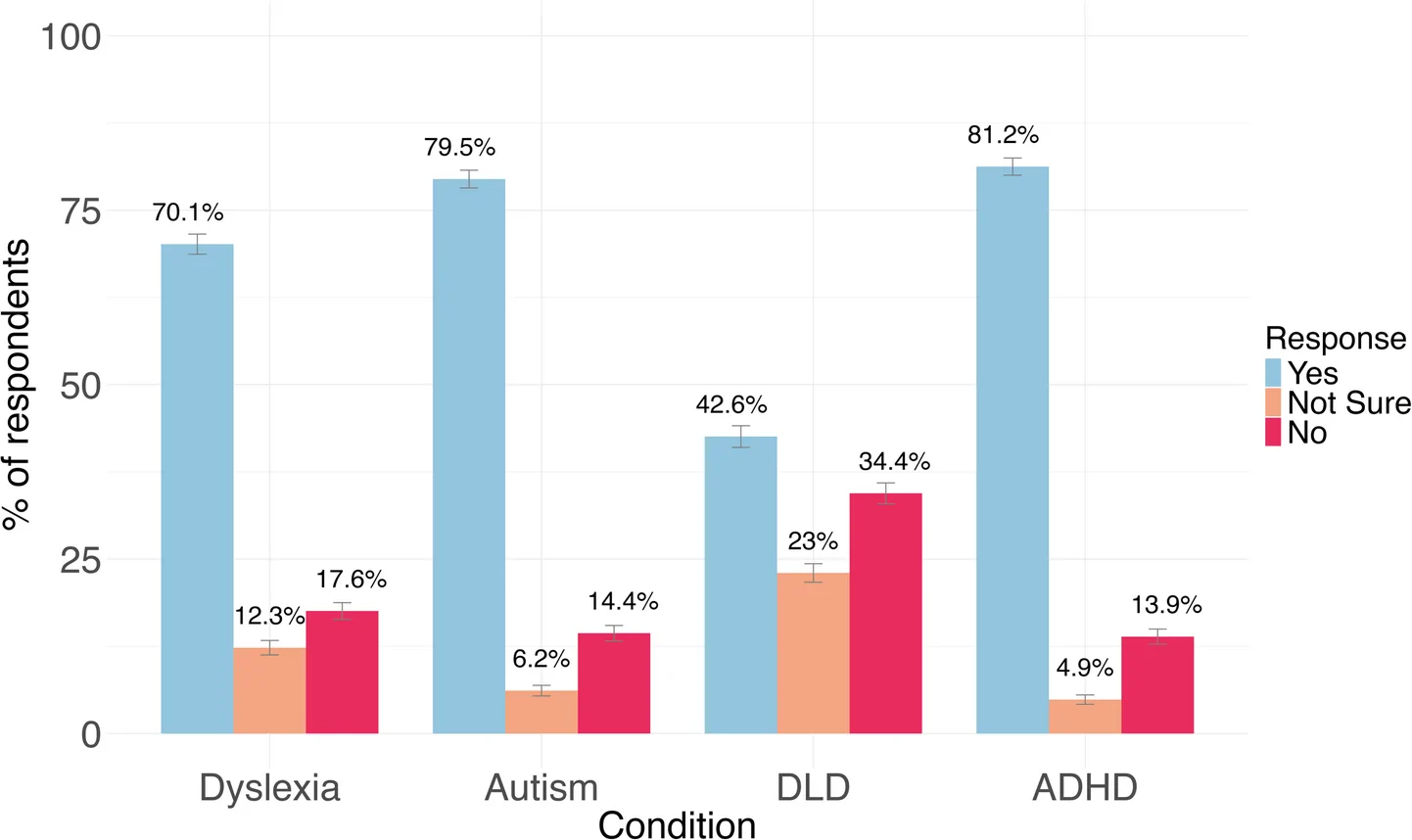
Respondents’ levels of awareness of dyslexia and other neurodevelopmental conditions

Figure 1 illustrates respondents’ awareness of dyslexia and several common neurodevelopmental conditions. Overall, 70.1% of respondents reported having heard of dyslexia. Awareness of dyslexia was lower than awareness of other conditions, with 81.2% reporting familiarity with ADHD and 79.5% with autism. However, awareness of dyslexia was higher than that of DLD, which was recognised by only 42.6% of respondents.

**Fig. 2:**
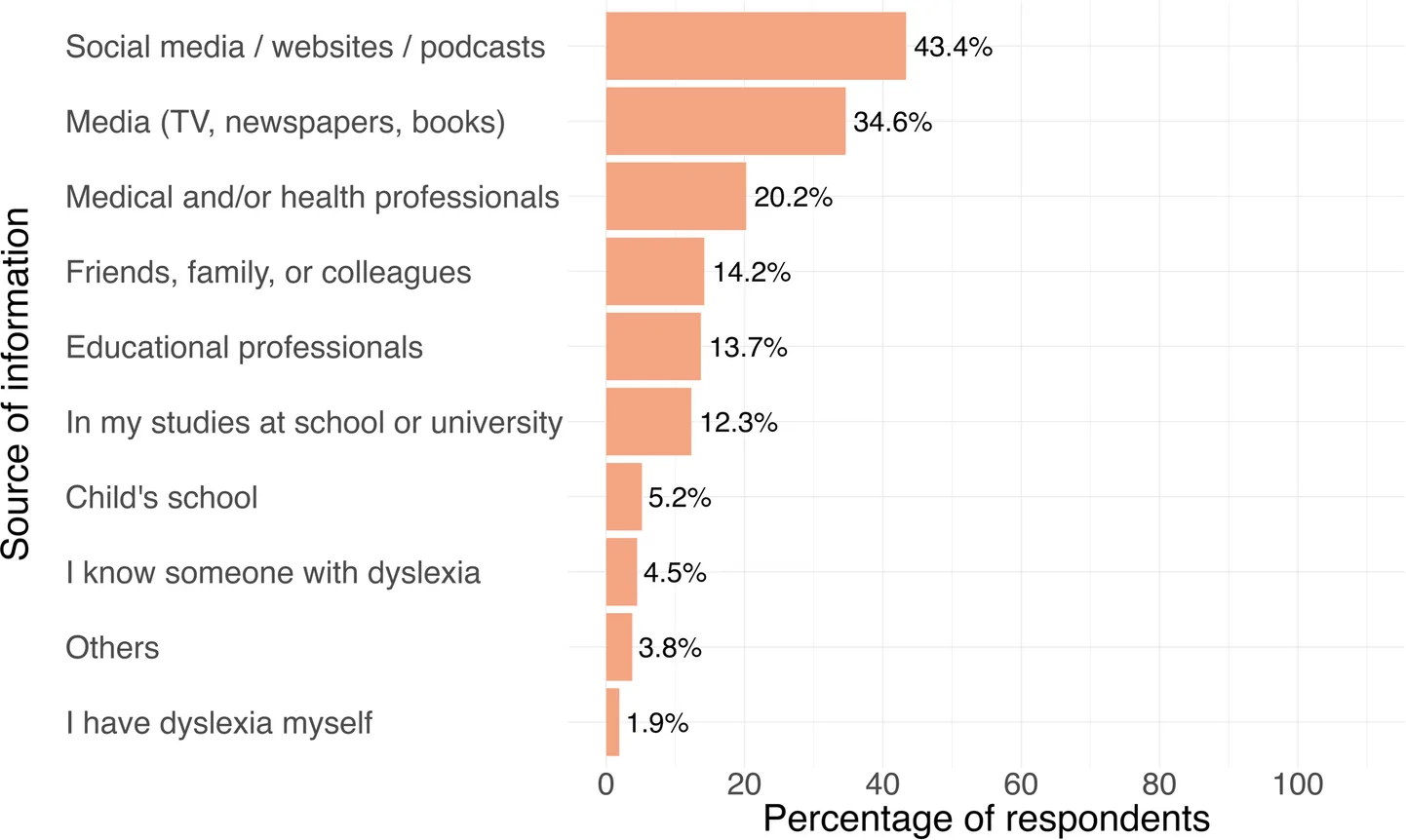
Reported sources of information about dyslexia

Figure 2 shows the most reported sources of information among respondents who had heard of dyslexia. In terms of common sources of information, around 61.8% reported hearing about it through social media, websites, or podcasts. This was followed by 49.4% who heard about it through traditional media sources such as TV, newspapers, or books, and 28.9% who reported learning about it from medical or health professionals.

Table 2 presents the level of awareness of dyslexia across demographic groups.

**Table 2:** Dyslexia awareness levels across demographic groups

Demographic Groups	Yes	No/Not Sure
**Age (years)**	% (*n*)	% (*n*)
18–29	79.4 (278)	20.6 (72)
30–39	66.0 (138)	34.0 (71)
40–49	69.6 (183)	30.4 (80)
50–59	59.3 (89)	40.7 (61)
60+	52.8 (19)	47.2 (17)
**Gender**		
Female	74.2 (431)	25.8 (150)
Male	64.2 (269)	35.8 (150)
**Area of residence**		
Rural	59.8 (76)	40.2 (51)
Urban	71.6 (631)	28.4 (250)
**Geographical region**		
East China	70.7 (263)	29.3 (109)
North China	78.0 (78)	22.0 (22)
Northeast China	67.0 (126)	33.0 (62)
Northwestern China	73.9 (113)	26.1 (40)
South Central China	62.9 (100)	37.1 (59)
Southwestern China	75.0 (27)	25.0 (9)
**Education level**		
Primary school	54.3 (19)	45.7 (16)
Secondary/vocational school	59.4 (123)	40.6 (84)
College/bachelor’s degree	72.5 (456)	27.5 (173)
Postgraduate degrees	81.2 (108)	18.8 (25)
**Income level**		
Low income	63.7 (158)	36.3 (90)
Middle income	71.1 (256)	28.9 (104)
High income	78.2 (158)	21.8 (44)
**Parental status**		
Non-parents	76.2 (320)	23.8 (100)
Parents	65.8 (387)	34.2 (201)

Age group was significantly associated with dyslexia awareness, χ² (4) = 29.70, *p *< .001. Post hoc comparisons revealed that participants aged 18-29 years were significantly more likely to have heard of dyslexia than those aged 30-39 years (*p* < .05), 50-59 years (*p* < .05), and 60+ years (*p* < .05), with no significant differences between other age groups. There was a significant association between gender and dyslexia awareness, χ² (1) = 11.55, *p *< .001, with 74.2% of females having heard of dyslexia compared to 64.2% of males. Urban residents (71.6%) were significantly more likely to report awareness of dyslexia than rural residents (59.8%), χ² (1) = 7.36, *p* = .007. In contrast, geographical region (χ² (5) = 9.28, *p* = .098) was not significantly associated with dyslexia awareness. Education level showed a significant association with awareness levels, χ² (3) = 25.07, *p* < .001. Post hoc comparisons revealed that 81.2% of respondents with postgraduate degrees were significantly more likely to have heard of dyslexia compared to those with secondary (59.4%, *p* < .05) and primary school education (54.3%, *p *< .05). Awareness of dyslexia was also significantly more likely among those with bachelor’s degrees (72.5%) than those with secondary education only (*p* < .05). In addition, income level was significantly associated with dyslexia awareness, χ² (2) = 11.37, *p* = .003, with respondents in the high-income group (78.2%) being more likely to report awareness than those in the low-income group (63.7%, *p* < .05). Finally, non-parents (76.2%) were significantly more likely than parents (65.8%) to be aware of dyslexia, χ² (1) = 12.59, *p* < .001.

### Dyslexia knowledge

On average, respondents answered 49% (*SD* = 20.51) of the statements correctly. For each area of knowledge assessed (causes and prevalence, symptoms, functional impact/outcomes, assessment and treatment), Table 3 summarises each statement with the percentage of respondents who selected “True”, “False”, or “Unsure”, as well as the correct answer for each statement.

**Table 3:** Percentage distribution of responses to dyslexia knowledge statements

Statements	TRUE	FALSE	Unsure
	% (*n*)	% (*n*)	% (*n*)
**Causes and Prevalence**			
1. Dyslexia is rare in Chinese speakers.	34.6 (349)	**25.6 (258)**	39.8 (401)
2. Dyslexia is a brain-based disorder.	**61.1 (616)**	8.0 (81)	30.9 (311)
3. Dyslexia is caused by poor parenting style or lack of discipline.	30.7 (309)	**35.2 (355)**	34.1 (344)
4. Dyslexia only affects children but not adults.	14.7 (148)	**64.2 (647)**	21.1 (213)
5. Dyslexia occurs because a child is not putting enough effort into learning to read.	21.9 (221)	**56.5 (570)**	21.5 (217)
6. Dyslexia runs in families (it is genetic).	**41.9 (422)**	11.3 (114)	46.8 (472)
7. Dyslexia is more frequent in males than in females.	**22.4 (226)**	13.8 (139)	63.8 (643)
8. Dyslexia occurs because of intellectual difficulties.	18.7 (188)	**43.2 (435)**	38.2 (385)
9. Dyslexia occurs due to insufficient/poor reading instruction.	31.7 (320)	**32.9 (332)**	35.3 (356)
10. Dyslexia occurs due to laziness.	15.5 (156)	**63.5 (640)**	21.0 (212)
**Symptoms**			
11. Children with dyslexia tend to have difficulties with spelling.	**77.3 (779)**	5.3 (53)	17.5 (176)
12. Difficulties producing clear, legible handwriting.	**41.4 (417)**	20.9 (211)	37.7 (380)
13. Frequently mix up Chinese characters that look the same, for example, 人(ren2; rén) and 入 (ru4; rù) when reading.	**57.5 (580)**	10.3 (104)	32.1 (324)
14. Generally, children with dyslexia have problems with phonological awareness (e.g., the ability to hear and manipulate sounds in language).	**53.6 (540)**	11.6 (117)	34.8 (351)
15. Individuals with dyslexia may struggle with understanding what they read.	**79.5 (801)**	5.2 (52)	15.4 (155)
16. Poor structure and organisation of written work.	**65.3 (658)**	7.6 (77)	27.1 (273)
17. Students with dyslexia often read slowly and lack of fluency.	**78.3 (789)**	4.8 (48)	17.0 (171)
18. Takes longer than others to complete written tasks.	**75.6 (762)**	4.2 (42)	20.2 (204)
**Functional Impact and Outcomes**			
19. Dyslexia can increase odds of living in poverty.	**65.8 (663)**	11.0 (111)	23.2 (234)
20. Dyslexia can affect negatively the behaviour and social-emotional well-being in affected individuals.	**71.2 (718)**	9.0 (91)	19.7 (199)
21. Dyslexia can increase odds of engaging in criminal behaviour.	**34.4 (347)**	22.7 (229)	42.9 (432)
22. Have an increased risk of showing behavioural difficulties.	**65.9 (664)**	10.8 (109)	23.3 (235)
23. May not enjoy reading.	**62.8 (633)**	12.0 (121)	25.2 (254)
24. Dyslexia resolves in childhood.	15.7 (158)	**35.3 (356)**	49.0 (494)
25. Students with dyslexia may show low academic performance.	**52.3 (527)**	14.6 (147)	33.1 (334)
26. Many students with dyslexia have low self-esteem.	**71.4 (720)**	5.6 (56)	23.0 (232)
27. Dyslexic students cannot succeed in their study and work.	26.9 (271)	**42.8 (431)**	30.4 (306)
**Assessment and Treatment**			
28. Multisensory instruction has been shown to be an ineffective teaching method for treating dyslexia.	40.7 (410)	**30.9 (311)**	28.5 (287)
29. Techniques involving repeated reading of material help to improve reading fluency.	**68.1 (686)**	8.6 (87)	23.3 (235)
30. Students with dyslexia need structured, sequential, direct instruction in basic skills and learning strategies.	**76.3 (769)**	3.6 (36)	20.1 (203)
31. Children with dyslexia can be helped by using coloured lenses/coloured overlays.	35.9 (362)	**10.3 (104)**	53.8 (542)
32. Medications can cure dyslexia.	22.8 (230)	**22.4 (226)**	54.8 (552)
33. Eye tracking exercises are effective in remediating dyslexia-caused difficulties.	49.7 (501)	**6.6 (67)**	43.7 (440)
34. Dyslexia improves with a positive attitude or effort.	63.7 (642)	**8.3 (84)**	28.0 (282)
35. Dyslexia students benefit from extra exam time.	**43.3 (436)**	21.6 (218)	35.1 (354)
36. Dyslexia can be completely cured.	31.5 (318)	**16.4 (165)**	52.1 (525)
37. Dyslexia can be resolved with hard work.	51.5 (519)	**14.5 (146)**	34.0 (343)
38. Formal testing of reading, writing and language is necessary to diagnose dyslexia.	**71.7 (723)**	4.6 (46)	23.7 (239)

Percentages of correct responses are highlighted in bold

In terms of knowledge about the causes and prevalence of dyslexia, over 60% of respondents correctly identified that dyslexia does not only affect children and is not caused by laziness or a lack of effort. However, less than half correctly recognised that dyslexia is not related to intellectual difficulties, and just 41.9% understood it as a genetic disorder. Fewer than 40% accurately indicated that dyslexia is not caused by poor parenting or inadequate teaching, that it is not rare among speakers of Chinese, or that it is more prevalent among males, with the majority either showing inaccurate or limited knowledge. In terms of knowledge of dyslexia symptoms, over 70% correctly recognised that individuals with dyslexia may struggle with understanding what they read, tend to have difficulties with spelling, often read slowly and lack fluency, and may take longer than others to complete written tasks. Over 60% also correctly recognised that poor structure and organisation of written work can be a feature of dyslexia. Just over half of the respondents correctly identified that children with dyslexia may frequently mix up Chinese characters that look similar or that they generally have problems with phonological awareness. Only 41.4% of respondents recognised that dyslexia may be associated with handwriting difficulties, with the majority showing lack of or inaccurate knowledge. In terms of knowledge about the functional impact and outcomes of dyslexia, around 70% of respondents correctly identified that dyslexia can negatively affect social-emotional wellbeing, is associated with low self-esteem, and can increase the risk of behavioural difficulties. Similarly, over 60% recognised that dyslexia may increase the odds of living in poverty and that individuals with dyslexia may not enjoy reading. However, only 52.3% correctly identified that students with dyslexia may show low academic performance. Misconceptions were evident. Only 42.8% correctly recognised that students with dyslexia can succeed in their studies and work, and just 35.3% acknowledged that dyslexia does not resolve in childhood. Around 57.2% of respondents either held inaccurate knowledge or lacked knowledge regarding individuals with dyslexia’s ability to succeed academically and professionally. Similarly, 64.7% did not recognise that dyslexia is a lifelong condition, with nearly half (49%) indicating a lack of knowledge. Only 34.4% identified the potential link between dyslexia and an increased risk of criminal behaviour, with the majority reporting lack of knowledge. With regards to dyslexia assessment and intervention, around 70% of respondents correctly recognised the importance of structured, sequential instruction, the need for formal testing to diagnose dyslexia, and the value of repeated reading for improving reading fluency. However, less than half of the respondents understood that students with dyslexia benefit from extra exam time, and only 30.9% correctly identified that multisensory instruction is effective. Only 22.4% correctly identified that medications cannot cure dyslexia, and just 16.4% rejected the idea that dyslexia can be completely cured. Similarly, 85.5% either had incorrect knowledge or were unsure whether dyslexia could be resolved through hard work. The use of coloured overlays and eye tracking exercises was also misunderstood, with only 10.3% and 6.6% correctly identifying these as ineffective, respectively. 

Analysis using one-way ANOVA revealed significant differences in dyslexia knowledge scores across the different areas of knowledge assessed (*F* (2.41, 2428.67) = 687.00, *p* <.001, *η*² = 0.41). Overall, respondents had better knowledge of dyslexia symptoms (*M* = 66.05%, *SD* = 30.72), followed by functional impact/outcomes (*M* = 55.76%, *SD* = 27.06) and causes and prevalence (*M* = 44.65%, *SD* = 25.64), with the lowest accuracy being for statements on dyslexia assessment and treatment (*M* = 33.52%, *SD* = 17.49). Bonferroni-adjusted post hoc comparisons showed that all pairwise differences were statistically significant (*p* <.001).

Age had a significant main effect on dyslexia knowledge scores, Welch’s *F* (4, 208.47) = 6.64, *p* <.001, *η*² = 0.03, 95% CI [0.01, 0.04]. On average, the highest scores were observed among participants aged 18–29 (*M* = 52.85%, *SD* = 18.66), followed by those aged 50–59 (*M* = 47.44%, *SD* = 20.22), 30–39 (*M* = 47.36%, *SD* = 19.36), 60+ (*M* = 45.03%, *SD* = 23.71), and 40–49 (*M* = 44.96%, *SD* = 22.50). Post hoc Tukey comparisons indicated that the 18–29 group scored significantly higher than both the 30–39 (*p* =.017) and 40–49 (*p* <.001) groups. No other pairwise comparisons reached statistical significance. Moreover, knowledge scores differed significantly according to gender, *t* (998) = 4.56, *p <*.001, Cohen’s *d* = 0.29, 95% CI [0.17, 0.42], with females’ knowledge scores (*M* = 51.12%, *SD* = 19.83) higher than males’ scores (*M* = 45.18%, *SD* = 21.00). Urban residents’ dyslexia knowledge scores (*M* = 49.24%, *SD* = 20.26) were also significantly higher than those of rural residents (*M* = 43.93%, *SD* = 21.66), *t* (1006) = 2.74, *p* =.006, Cohen’s *d* = 0.26, 95% CI [0.07, 0.45]. The effect of geographical region was significant as well, *F* (5, 1002) = 3.19, *p* =.007, *η*² = 0.02, 95% CI [0.00, 0.03]. Residents from East China (*M* = 50.43%, *SD* = 20.59) and Southwestern China (*M* = 55.19%, *SD* = 20.10) scored significantly higher than those from Northeast China (*M* = 44.36%, *SD* = 20.86), with *p* =.012 and *p* =.042 respectively. The pairwise comparisons among North China (*M* = 49.45%, *SD* = 21.92), Northwestern China (*M* = 47.04%, *SD* = 20.18), and South Central China residents (*M* = 48.59%, *SD* = 18.58) were nonsignificant. Furthermore, education level had a significant main effect on knowledge scores, *F* (3, 1000) = 14.15, *p <*.001, *η*² = 0.04, 95% CI [0.02, 0.07]. Respondents with postgraduate (*M* = 52.43%, *SD* = 20.17) or bachelor’s (*M* = 50.27%, *SD* = 19.82) degrees had the highest mean knowledge scores, significantly outperforming those with only secondary education (*M* = 43.94%, *SD* = 20.26), *p <*.001, respectively. Additionally, participants with only primary education (*M* = 32.63%, *SD* = 23.34) scored significantly lower than all other groups (*p* <.001 to *p* =.001). No significant difference was found between the bachelor’s and postgraduate groups (*p* =.671). Income level also significantly influenced dyslexia knowledge, *F* (2, 807) = 17.85, *p* <.001, *η*² = 0.04, 95% CI [0.02, 0.07]. High-income respondents’ (*M* = 55.12%, *SD* = 19.59) mean knowledge score was the highest, followed by middle- (*M* = 50.04%, *SD* = 19.63) and low-income groups (*M* = 43.94%, *SD* = 20.54). All pairwise differences related to income level were significant (*p* <.001 to *p* =.001). Finally, knowledge scores differed significantly according to parental status, as parents (*M* = 46.84%, *SD* = 20.82) had significantly lower accuracy scores than non-parents (*M* = 50.98%, *SD* = 19.83), *t* (928.02) = 3.20, *p =*.001, Cohen’s *d* = 0.20, 95% CI [0.08, 0.33].

### Attitudes towards dyslexia

**Fig. 3:**
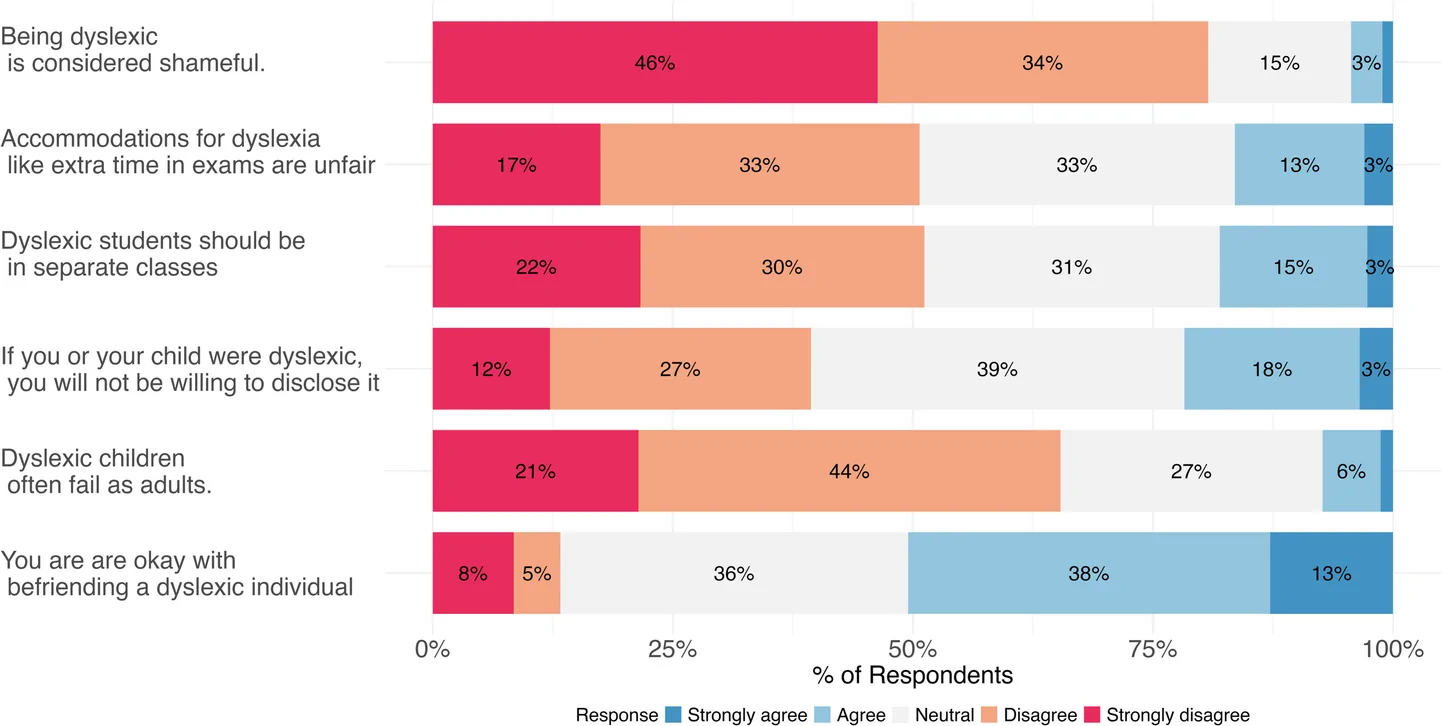
Respondents’ levels of agreement with attitude-related statements

As shown in Fig. 3 and 80% of respondents strongly disagreed or disagreed that having dyslexia is shameful. Around 65% strongly disagreed or disagreed that children with dyslexia will fail in the future. Around 51% agreed or strongly agreed that they are okay with befriending an individual with dyslexia. At least half of the respondents disagreed or strongly disagreed that giving extra time in exams for dyslexic students is unfair, or that dyslexic students should be put in separate classes, with the remaining proportions either reporting feeling neutral or agreeing with these statements. Around 39% of respondents strongly disagreed or disagreed that if they or their child had dyslexia, they would not disclose it.

Table 4 presents the descriptive statistics regarding respondents’ attitude scores across different demographic groups. Dyslexia attitude scores significantly differed by age, *F* (4, 1003) = 5.31, *p <*.001, *η*² = 0.02, 95% CI [0.01, 0.04]. Respondents aged 18–29 had significantly higher attitude scores than those aged 30–39, 40–49, 50–59, and 60+ (all *p*-values < 0.05), with all other pairwise differences being nonsignificant. However, gender did not have a significant effect on attitudes towards dyslexia, *t* (800.00) = 1.25, *p* =.211, Cohen’s *d* = 0.08, 95% CI [−0.04, 0.21]. The effect of area of residence was significant, *t* (1006) = 3.27, *p* =.001, Cohen’s *d* = 0.31, 95% CI [0.12, 0.50], with urban residents reporting significantly more positive attitudes than rural residents. Similarly, a significant effect of region on dyslexia attitude scores was found, *F* (5, 1002) = 3.80, *p* =.002, with a small effect size (*η*² = 0.02, 95% CI [0.00, 0.03]). Respondents from Northeast China reported significantly lower attitude scores than the East China (*p* =.001) and Northwestern China counterparts (*p* =.009). No other regional comparisons were significant. In addition, dyslexia attitude scores differed according to education level, *F* (3, 1000) = 7.30, *p* <.001, *η*² = 0.02, 95% CI [0.01, 0.04]. Respondents with only primary and secondary education expressed significantly less positive attitudes than those with bachelor’s degrees (primary vs. bachelor’s *p* =.032; secondary vs. bachelor’s *p* =.014) and those with postgraduate education (primary vs. postgraduate *p* =.004; secondary vs. postgraduate *p* =.001). Moreover, income level also significantly affected attitudes towards dyslexia, Welch’s *F* (2, 466.44) = 17.66, *p <*.001, *η*² = 0.05, 95% CI [0.02, 0.08]. All pairwise differences were significant (all *p-*values < 0.05). Finally, parents’ attitudes towards dyslexia were significantly less positive than non-parents, *t* (1006) = 4.57, *p <*.001, Cohen’s *d* = 0.29, 95% CI [0.17, 0.42].

**Table 4:** Respondents’ average attitude scores across demographic groups

Demographic Groups	Mean	Standard Deviation
**Age (years)**		
18–29	22.33	3.63
30–39	21.32	3.48
40–49	21.52	3.22
50–59	21.23	3.24
60+	20.61	3.73
**Gender**		
Female	21.81	3.21
Male	21.52	3.83
**Area of residence**		
Rural	20.75	3.72
Urban	21.82	3.42
**Geographical region**		
East China	22.00	3.38
North China	21.71	3.76
Northeast China	20.77	3.59
Northwestern China	22.05	3.62
South Central China	21.55	3.02
Southwestern China	22.19	3.50
**Education level**		
Primary school	20.20	4.14
Secondary/vocational school	21.00	3.53
College/bachelor’s degree	21.84	3.34
Postgraduate degrees	22.42	3.57
**Income level**		
Low income	20.74	3.72
Middle income	21.95	3.17
High income	22.66	3.22
**Parental status**		
Non-parents	22.27	3.51
Parents	21.27	3.38

### Relationship between dyslexia awareness, knowledge, and attitudes

The association between dyslexia awareness and knowledge was significant, *t* (529.36) = 6.62, *p* <.001, Cohen’s *d* = 0.47, 95% CI [0.33, 0.61], with average knowledge accuracy higher among participants who were aware of dyslexia (*M* = 51.38%, *SD* = 19.58) than among those who were unaware/unsure (*M* = 41.96%, *SD* = 21.13). Respondents aware of dyslexia (*M* = 21.96, *SD* = 3.44) showed significantly more positive attitudes compared to those who were unaware/unsure (*M* = 21.05, *SD* = 3.47), *t* (1006) = 3.82, *p <*.001, Cohen’s *d* = 0.26, 95% CI [0.13, 0.40]. Additionally, a significant small-to-moderate positive correlation was found between dyslexia knowledge (*M* = 48.57%, *SD* = 20.51) and attitude (*M* = 21.69, *SD* = 3.47), *r* (1006) = 0.25, *p* <.001, 95% CI [0.19, 0.31].

Then, hierarchical multiple regressions determined if the associations remained after controlling for age, geographical region, area of residence, parental status, education, and income level. Regarding the dyslexia awareness-knowledge relationship, the initial model explained 7.1% of the variance in knowledge (*R*² = 0.07, *F* (6, 801) = 10.21, *p* <.001). The full model that added dyslexia awareness remained significant, *R*² = 0.10, *F* (7, 800) = 12.85, *p* <.001, with a significant increase in *R*² of 0.03 (*p* <.001). Dyslexia awareness significantly predicted knowledge (*b* = 7.94, *SE* = 1.54, β = 0.18, *p* <.001, 95% CI [4.93, 10.96]). Education (*b* = 3.25, *SE* = 1.11, β = 0.11, *p* =.003, 95% CI [1.07, 5.43]) and income (*b* = 3.81, *SE* = 1.00, β = 0.14, *p* <.001, 95% CI [1.86, 5.77]) were also significant unique predictors. For the dyslexia awareness-attitude relationship, the covariates alone explained 8.2% of the variance in attitudes (*R*² = 0.08, *F* (6, 801) = 11.98, *p* <.001). After adding dyslexia awareness into Step 2, the full model was significant, *F* (7, 800) = 10.63, *p* <.001. There was a non-significant increase in R² (*p* =.123). Only area of residence (*b* = −1.01, *SE* = 0.37, *β* = − 0.10, *p* =.006, 95% CI [−1.73, −0.29]) and income level (*b* = 0.88, *SE* = 0.17, *β* = 0.19, *p* <.001, 95% CI [0.55, 1.22]) remained as unique predictors in the final model. For the dyslexia knowledge-attitude relationship, the demographic covariates in Step 1 explained 8.2% of the variance in attitudes (*R*² = 0.08, *F* (6, 801) = 11.98, *p* <.001). The addition of dyslexia knowledge led to a significant full model, *F (*7, 800) = 14.60, *p* <.001, which explained 11.3% of variance (*R*² = 0.11). There was a significant R² increase of 0.03 (*p* <.001). In the final model, area of residence (*b* = −0.98, *SE* = 0.36, β = − 0.09, *p* =.007, 95% CI [−1.69, −0.27]), income level (*b* = 0.77, *SE* = 0.17, β = 0.17, *p* <.001, 95% CI [0.44, 1.10]), and dyslexia knowledge (*b* = 0.03, *SE* = 0.01, β = 0.18, *p* <.001, 95% CI [0.02, 0.04]) uniquely predicted attitudes towards dyslexia.

## Discussion

This study is the first to examine public awareness, knowledge, and attitudes towards dyslexia in Mainland China. Our findings indicate a relatively good level of dyslexia awareness among the Chinese public, along with broadly positive attitudes towards individuals with dyslexia. However, knowledge about dyslexia was limited. Levels of awareness, knowledge, and attitudes varied across demographic groups. Awareness was associated with higher knowledge, whereas knowledge (but not awareness) emerged as a predictor of attitudes towards dyslexia.

### There is a growing public awareness of dyslexia in China

Contrary to our hypothesis, the level of dyslexia awareness among the Chinese public was relatively high. Around 70% of respondents reported having heard of dyslexia. This figure should be interpreted with caution given that the composition of the study’s sample was skewed towards younger, more educated, and predominantly urban respondents. These demographic groups have consistently been shown to have higher levels of dyslexia awareness (Castillo & Gilger, 2018; Duranović et al., 2011; Furnham, 2013). Nevertheless, this level of awareness was lower than that reported in the USA (91%; Castillo & Gilger, 2018) and Croatia (88%). One possible explanation for this difference relates to variation in the national policy context. In the USA, dyslexia has been acknowledged in disability legislation for more than 55 years (Grigorenko et al., 2020). In contrast, and as noted by Yin et al. (2020), the first official policy document in China to mention dyslexia was not issued until 2014. This reflects a significant delay in governmental recognition of dyslexia in China.

Our findings further revealed that public awareness of dyslexia was lower than the awareness of other neurodevelopmental conditions, such as ADHD (81.2%) and autism (79.5%). This difference may reflect the greater behavioural visibility of autism and ADHD. In addition, autism received earlier policy recognition in China as it had been officially classified as a mental disability in 2009, followed by the introduction of support services (Luo et al., 2022) which may have contributed to greater public familiarity with autism.

Most participants who had heard of dyslexia reported learning about it through social and traditional media (e.g., TV, newspapers, and books), which emerged as the most common sources of information. This pattern is consistent with findings from the Balkan countries (Duranović et al., 2011). This also reflects recent trends in the use of digital platforms to access health-related information (Mather et al., 2020). In contrast, fewer participants reported hearing about dyslexia through medical or educational professionals, which could potentially be because media platforms are more easily accessible and often free of charge.

Awareness of dyslexia in this study was significantly associated with age, gender, and education level. Participants aged 18–29 years had higher awareness of dyslexia relative to the other age groups. This result is in line with previous studies which found higher dyslexia awareness among younger adults (Duranović et al., 2011; Castillo & Gilger, 2018). Younger adults are likely spending more time on social media, which emerged as the most common source of information in this sample. Higher awareness was also reported among females and participants with more years of formal education, consistent with findings from Western contexts (Furnham, 2013; Castillo & Gilger, 2018). In our sample, women were over-represented relative to national census data. This may have contributed to the overall awareness levels observed and may also reflect gender differences in engagement with learning and help-seeking behaviours.

In many households, women are expected to take primary responsibility for children’s upbringing and education (Zou et al., 2022). In fact, prior studies showed that Chinese mothers tend to be more actively involved in their children’s education and are more engaged in seeking information about learning difficulties (Sun et al., 2013). The traditional social dynamics in China may partly explain the higher levels of awareness among females in this study. The association between higher education and greater awareness may be linked to increased exposure to social and developmental topics among those with higher levels of education (Duranović et al., 2011). By contrast, geographical region was not significantly associated with dyslexia awareness. This finding should be interpreted cautiously, as respondent numbers across geographical regions were uneven, and in some cases (e.g., Southwestern China), this may have resulted in insufficient statistical power to capture small effects.

### A significant gap in the public knowledge of dyslexia

We predicted that public knowledge of dyslexia in Mainland China would be limited, with better recognition of behavioural symptoms but weaker understanding of its causes, treatment, and long-term impact (Furnham, 2013; Castillo & Gilger, 2018; Yin et al., 2020). The findings of this study are largely consistent with these predictions.

On average, respondents answered only 49% of the knowledge statements correctly, indicating an overall insufficient understanding of dyslexia. Most recognised dyslexia as a brain-based condition affecting both children and adults and rejected statements attributing it to laziness or lack of effort. This pattern was also observed in the USA, where neurological explanations were commonly endorsed by the public (Castillo & Gilger, 2018). However, some uncertainty and misconceptions about dyslexia’s causes and prevalence were evident. Less than half of the respondents recognised the genetic basis of dyslexia, with the majority reporting uncertainty, which mirrored findings from the UK (Furnham, 2013). Similarly, just above 40% of respondents rejected the statement that dyslexia is related to low intelligence, with the majority expressing uncertainty, and a further 19% endorsing the statement. This misconception was also observed in between 8% and 23% across the Balkan countries (Duranović et al., 2011). Moreover, almost one-third attributed dyslexia to poor parenting and lack of discipline, or insufficient reading instruction. These findings are consistent with the strong cultural emphasis in China on the role of parents and teachers in determining children’s academic success (Li, 2012), which may lead to the perception that learning difficulties result from inadequate parent or teacher practices. Only 26% rejected the statement that dyslexia is rare among Chinese speakers, consistent with the previously widespread belief that dyslexia did not exist in Chinese (McBride et al., 2018).

Consistent with findings from Western contexts, respondents in our study demonstrated relatively good knowledge of the core symptoms of dyslexia, including reading and spelling difficulties. However, knowledge of phonological and handwriting difficulties was more limited, likely because these difficulties are less visible than reading problems.

In terms of functional impact, most recognised that dyslexia can negatively affect social-emotional wellbeing, self-esteem, behaviour, and reading enjoyment, and that it may be associated with an increased risk of poverty. However, understanding of academic and long-term outcomes was more limited. Only around half of respondents recognised that students with dyslexia may demonstrate low academic performance, and just 43% recognised that individuals with dyslexia can succeed academically and professionally, and fewer than 36% correctly identified dyslexia as a lifelong condition. These findings mirror those of Castillo & Gilger (2018), who reported uncertainty and reduced confidence in long-term outcomes among Asian participants in the US. Reduced expectations regarding the academic and occupational potential of individuals with dyslexia have also been documented in Western populations (e.g., Macdonald, 2010).

Knowledge of dyslexia assessment and intervention emerged as the weakest domain. Most respondents recognised the need for formal assessment to diagnose dyslexia; however, fewer than half understood that students with dyslexia benefit from accommodations such as extra exam time. Misconceptions were evident as illustrated by the respondents’ endorsement of interventions or support practices that are not evidence-based, including coloured overlays, eye-tracking exercises, and medication. A large majority endorsed effort-based beliefs, with over 80% believing that dyslexia can be overcome through hard work and/or a positive attitude. Such beliefs are consistent with the Confucian emphasis on effort over ability (Li, 2012; Rao et al., 2000).

Direct comparison of overall dyslexia knowledge across countries is difficult, as knowledge was measured differently across studies. The present study used binary scoring (correct/incorrect), whereas UK and US studies relied on Likert-type agreement scales. Nevertheless, some misconceptions appear consistent across contexts, particularly regarding causes and controllability, with effort-based beliefs appearing especially prominent in the Chinese sample.

Participants aged 18–29 had the highest knowledge scores, followed by those aged 50–59. Higher scores among younger adults likely reflect greater exposure to dyslexia-related content through social media, the most commonly reported source for dyslexia information in this study. Non-parents had higher knowledge scores than parents, partly due to sample composition, as many non-parents were aged 18–29. Females demonstrated greater knowledge than males, consistent with previous findings (Furnham, 2013). Knowledge levels also differed by area of residence and geographical region, possibly reflecting differences in access to dyslexia-related information and services.

### Largely positive public attitudes towards dyslexia

Contrary to our predictions, participants in this study expressed generally positive attitudes towards dyslexia. The majority of respondents rejected negative stereotypes, with around 80% disagreeing that having dyslexia is shameful and approximately two-thirds rejecting the belief that children with dyslexia will fail in the future. Despite these positive views, a substantial proportion of participants expressed reluctance or uncertainty around disclosure, indicating potential fear of societal stigma. Importantly, more positive attitudes were observed among groups that also demonstrated higher levels of dyslexia awareness and knowledge, including younger respondents and those with higher levels of education and income.

### Dyslexia knowledge but not awareness predicts attitudes

Greater awareness of dyslexia was associated with higher knowledge scores, with respondents who had heard of dyslexia demonstrating greater understanding than those who were unaware or unsure. However, it is important to note that awareness does not equate to accurate knowledge. This is illustrated by the fact that around 70% of respondents reported having heard of dyslexia, yet, on average, they answered only around half of the knowledge statements correctly.

Awareness did not independently predict attitudes towards dyslexia once demographic variables were accounted for. In contrast, dyslexia knowledge emerged as a significant predictor of attitudes, with higher knowledge scores associated with more positive attitudes. This pattern is consistent with evidence suggesting that accurate knowledge and understanding of disability, rather than familiarity with diagnostic labels alone, is more strongly related to inclusive attitudes and behaviours (Burke & Sutherland, 2004; Scior & Werner, 2015).

### Implications

This study is the first empirical investigation into public awareness and knowledge of dyslexia in China, and provides a valuable foundation for future research, policy development, and public campaigns. A key implication is the need for dyslexia awareness campaigns that go beyond promoting familiarity with the term “dyslexia”, and that should seek to actively improve understanding of its causes, manifestations, and impact on learning and development. Improving the public’s understanding of dyslexia is essential for reducing misconceptions, facilitating earlier access to assessment and support services, and promoting more positive, inclusive attitudes towards children and young people with dyslexia.

Social and traditional media emerged as the most frequently reported sources of information on dyslexia. These platforms could be powerful tools for dissemination; however, it is concerning that despite high levels of exposure through these channels, participants’ actual knowledge of dyslexia was limited. This suggests that some online information may be inaccurate or not based on evidence. Notably, participants reported rarely accessing information from health or education professionals. Involving these professionals in the development of accessible, evidence-based online resources and materials could help ensure the public receives accurate information on dyslexia. Finally, campaigns should address barriers to disclosure linked to fear of judgement or stigma through emphasising the benefits of early identification and providing clear guidance on where and how to seek effective support, which could help families and individuals access timely support.

### Limitations

Although the sample in our study included participants from all regions, there was evidence of sampling bias, with some regions more heavily represented than others. Individuals from rural areas, those with lower levels of education, and those from lower socioeconomic backgrounds were under-represented, whereas females and younger age groups were over-represented. As a result, the sample did not fully mirror national census distributions, limiting the generalisability of the findings to the wider population. This imbalance may reduce variability in the data, constraining the detection of group differences, and likely inflate the levels of dyslexia awareness, particularly as younger and more educated participants reported higher levels of awareness. These sampling limitations were linked to the use of an online survey, which restricted participation among groups with limited access to technology or with lower digital literacy, such as older individuals and those living in rural or remote regions.

### Future research directions

Future research should adopt stratified sampling to ensure representation across China’s diverse regions and demographic groups. Combining online with offline (paper-and-pencil surveys) data collection methods could help reach older groups and those with limited digital literacy or access to technology.

Beyond measuring public awareness, future research should explore the lived experiences of individuals with dyslexia, including children, adults, and parents. This includes examining how Confucian values and stigma may influence attitudes toward diagnosis and disclosure, and how these factors affect access to support and services. Understanding these experiences can guide more culturally relevant awareness campaigns and inclusive policies.

The study revealed low awareness of DLD, with only 42% of participants familiar with the condition despite its high prevalence (affecting two children in every classroom) and frequent co-occurrence with dyslexia. Future research should examine public understanding of DLD in China, as raising awareness of this lifelong and under-recognised condition is crucial for improving early identification and support for children with language and literacy difficulties.

## Supplementary Information

Below is the link to the electronic supplementary material.

Supplementary Material 1 (DOCX 37.6 KB)

## Data Availability

All data files and analyses outputs can be found on the Open Science Framework via the link: https://osf.io/5wt7a/overview?view_only=d83c0bdd91184bbbab7f9713065dbc1a.
