# Supplementary material for: Public awareness, knowledge and attitudes towards dyslexia in China
Source: Ann Dyslexia. 2026 Jun 22;76(3):760–85. doi: 10.1007/s11881-026-00377-y (PMC13759581; doi:10.1007/s11881-026-00377-y)
Supplement: Supplementary file 1 [file 11881_2026_377_MOESM1_ESM.docx]

**Appendix A: Online Survey**

**Part 1: Demographic information**

1. **How old are you?**

- 18-29
- 30-39
- 40-49
- 50-59
- 60-69
- 70+

2. **What is your gender?**

- Male
- Female
- Prefer not to say

3. **Area of residence?**

- Anhui
- Beijing
- Chongqing
- Fujian
- Gansu
- Guangdong
- Guangxi
- Guizhou
- Hunan
- Hubei
- Henan
- Hebei
- Hainan
- Heilongjiang
- Jiangsu
- Jiangxi
- Jilin
- Liaoning
- Neimenggu
- Ningxia
- Qinghai
- Shanghai
- Sichuan
- Shaanxi
- Shanxi
- Shandong
- Tianjin
- Xizang
- Xinjiang
- Yunnan
- Zhejiang

4. **Do you live in a:**

- Urban area
- Rural area

5. **Do you have children?**

- Yes
- No

6. **Are any of your children under 18 (if 5 is yes)**

- yes
- no

7. **Your highest level of education is:**

- Primary school
- Secondary school
- High school/Secondary vocational school
- Junior college/bachelor’s degree
- Postgraduate degree
- Others____

8. **What is your family’s per capita disposable income level? (If you don't know your exact income, please estimate.)**

- Low-income group (9,542 yuan per person)
- Lower-middle-income group (21,608 yuan per person)
- Middle-income group (33,925 yuan per person)
- Upper-middle-income group (53,359 yuan per person)
- High-income group (98,809 yuan per person) 6. Prefer not to say
- Prefer not to say

9. **What sector do you currently work in? (Please select one option that best describes your current role)**

- Education (e.g., teacher, administrator, researcher, trainer)
- Finance, Business, or Insurance (e.g., accountant, banker, analyst, business consultant, financial advisor)
- Medical, Health, or Social Care (e.g., doctor, nurse, therapist, social worker, caregiver, psychologist)
- Manufacturing or Engineering (e.g., factory worker, technician, engineer, production manager)
- Construction (e.g., builder, contractor, architect, civil engineer)
- Agriculture (e.g., farmer, agricultural scientist, fisheries worker, forestry worker)
- Travel, Sport, or Leisure (e.g., travel agent, tour guide, coach, athlete, event manager, hotel staff)
- Technology or IT (e.g., software developer, IT specialist, data analyst, )
- Creative Arts or Media (e.g., writer, artist, filmmaker, journalist, graphic designer)
- Retail, Hospitality, or Customer Service (e.g., shop assistant, cashier, waiter, hotel manager)
- Government or Public Service (e.g., policymaker, public servant, military personnel, law enforcement)
- Unemployed
- Retired
- Student
- Other (please specify sector): _______

**Part 2: Your awareness of dyslexia:**

10. **Have you heard about any of the following conditions:**

|  | Yes | No | Not Sure |
| --- | --- | --- | --- |
| Dyslexia |  |  |  |
| Autism |  |  |  |
| Developmental Language Disorder |  |  |  |
| ADHD |  |  |  |

**11. Only need to answer this question if the answer is ‘yes’ to dyslexia in Q10///Where did you hear about dyslexia before participating in this survey? You can choose more than one option**

- Medical and/or health professionals
- Educational professionals
- Child’s school
- In my studies at school or university
- Social media/Websites/Podcasts
- Media (e.g., TV, newspaper, books)
- Friends, family, or colleagues
- I know someone with dyslexia
- I have dyslexia myself
- Others ______

**12. Please tick all the difficulties or statements you think individuals with dyslexia may encounter in the table below.**

|  | True | False | Unsure |
| --- | --- | --- | --- |
| statement |  |  |  |
| 1. Dyslexia may increase the risk of living in poverty. |  |  |  |
| 2. Dyslexia is rare in Chinese speakers. |  |  |  |
| 3. Individuals with dyslexia may struggle with understanding what they read. |  |  |  |
| 4. Children with dyslexia tend to have more difficulties with spelling. |  |  |  |
| 5. Multisensory instruction (a teaching method that combines multiple senses, such as hearing, sight, touch, etc.) has been shown to be an ineffective teaching method for treating dyslexia. |  |  |  |
| 6. Dyslexia is a brain-based disorder. |  |  |  |
| 7. Techniques involving repeated reading of material help to improve reading fluency. |  |  |  |
| 8. Dyslexia is caused by poor parenting style or lack of discipline. |  |  |  |
| 9. Dyslexia can affect negatively the social-emotional well-being in affected individuals. |  |  |  |
| 10. Dyslexia can increase odds of engaging in criminal behaviour (to affected individuals). |  |  |  |
| 11. Frequently mix up Chinese characters that look the same, for example, 人 (ren2; rén) and 入 (ru4; rù) when reading. |  |  |  |
| 12. Students with dyslexia need structured, sequential, direct instruction in basic skills and learning strategies. |  |  |  |
| 13. Dyslexia only affects children but not adults. |  |  |  |
| 14. Students with dyslexia often lack accuracy and fluency when doing reading. |  |  |  |
| 15. Dyslexia may increase the risk of individuals experiencing behavioural difficulties (e.g., having trouble following classroom rules) |  |  |  |
| 16. Children with dyslexia can be helped by using coloured lenses/coloured overlays. |  |  |  |
| 17. Dyslexia occurs because a child is not putting enough effort into learning to read. |  |  |  |
| 18. Difficulties producing clear, legible handwriting. |  |  |  |
| 19. Medications can cure dyslexia. |  |  |  |
| 20. Eye tracking exercises are effective in remediating dyslexia-caused difficulties. |  |  |  |
| 21. Individuals with dyslexia may not enjoy reading |  |  |  |
| 22. Dyslexia runs in families (it is genetic). |  |  |  |
| 23. Dyslexia resolves in childhood. |  |  |  |
| 24. Dyslexia is more frequent in males than in females. |  |  |  |
| 25. Dyslexia improves with a positive attitude or effort. |  |  |  |
| 26. Dyslexia occurs because of intellectual difficulties. |  |  |  |
| 27. Generally, children with dyslexia have problems with phonological awareness (e.g., the ability to hear and manipulate sounds in language). |  |  |  |
| 28. Dyslexic students benefit from extra exam time. |  |  |  |
| 29. Dyslexia can be completely cured. |  |  |  |
| 30. Students with dyslexia may show low academic performance. |  |  |  |
| 31. Many students with dyslexia have low self -esteem |  |  |  |
| 32. Dyslexia occurs due to insufficient/poor reading instruction |  |  |  |
| 33. Dyslexic individuals may have poor language structure and organisation of written work. (e.g., grammatical mistakes and lack of organization in writing). |  |  |  |
| 34. Dyslexic students cannot succeed in their study and work. |  |  |  |
| 35. Dyslexia can be resolved with hard work. |  |  |  |
| 36. Formal testing of reading, writing and language is necessary to diagnose dyslexia. |  |  |  |
| 37. Dyslexia occurs due to Laziness. |  |  |  |
| 38. Dyslexic individuals need more time than others to complete written tasks |  |  |  |

**13. When do you think dyslexia can be diagnosed?**

- Before primary school
- Primary school/Secondary school
- Secondary vocational school/High school
- Over 18 years old
- All of above

**14. Who can diagnose dyslexia?**

- Class teachers
- Head teachers
- Qualified professionals (e.g., Educational psychologists, clinical psychologists)
- Developmental paediatricians
- Speech-language therapists
- Teaching assistants
- Parents and caregivers
- Special education teachers
- Literacy teachers (who teach students literacy and reading skills)

**15. Who can support dyslexic students? (Multiple choice)**

- Class teachers
- Head teachers
- Qualified professionals (e.g., Educational psychologists, clinical psychologists)
- Developmental paediatricians
- Speech-language pathologists
- Teaching assistants
- Parents and caregivers
- Special education teachers
- Literacy teachers (who teach students literacy and reading skills)

**Part 3: Your attitude towards dyslexia:**

| Statements | Strongly disagree | Disagree | Neutral | Agree | Strongly agree |
| --- | --- | --- | --- | --- | --- |
| 1. You are happy to have yourself or your children/students become friends with someone diagnosed with dyslexia. |  |  |  |  |  |
| 2. Dyslexic children often do not succeed as adults. |  |  |  |  |  |
| 3. If you or your child were diagnosed with dyslexia, you will not be willing to disclose the diagnosis to family, teachers or classmates. |  |  |  |  |  |
| 4. Students with dyslexia should not receive education together with other children. |  |  |  |  |  |
| 5. Giving students with dyslexia accommodations (like extra time in exams) is unfair to other students. |  |  |  |  |  |
| 6. Having dyslexia is considered shameful. |  |  |  |  |  |
